# Human digital twins in personalized and predictive healthcare: a comprehensive review of technologies, applications, and future directions

**DOI:** 10.3389/fdgth.2026.1827007

**Published:** 2026-06-19

**Authors:** A. Mohan Babu, E. S. Madhan

**Affiliations:** School of Computer Science and Engineering, Vellore Institute of Technology, Vellore, India

**Keywords:** artificial intelligence, computerized cardiology, digital twins, in silico medicine, personalized medicine, predictive healthcare

## Abstract

The role of real-time data, artificial intelligence, and computational modeling is discussed in this review analytics Human Digital Twins (HDTs) creation- virtual persons of personalities patients which advocate predictive simulation to forecast of physiological behavior, treatment responses, and disease tracks. A synthesis of existing knowledge is done up to the technologies is a foundation to HDTs, clinical application and implementation issues of interest to precision medicine. The conceptual basis of engineering of the digital twins is analyzed and production principles, and technologies, which allow to produce HDTs-machine. Are physiological modeling, learning and distributed cloud-based computing infrastructure identified and evaluated. Cards: cardiology, oncology, genomics and immunology are critically appraised. It is based on the comparative analysis of 35 peer-reviewed documents and technical as it was reported, HDTs have great potential in enhancing personalized prediction of side effects, optimization of clinical trial design using virtual, and scheduling of treatment cohort simulation. But, model standards, an important component of model validation, are not present interoperability, ethical governing mechanisms and regulatory avenues to clinical deployment. The main priority research directions are determined, such as the development of common-validation techniques; implementation of federated learning frameworks to support sharing of data with data privacy limitations; incorporation of multi-omics data into physiological models; and introducing open ethical review procedures. This review provides substantive evidence basis to researchers, clinicians and policy makers to market the. Knowledge about HDTs technology to population health and health care provision revolutionizes.

## 1 Introduction

**Figure 1:**
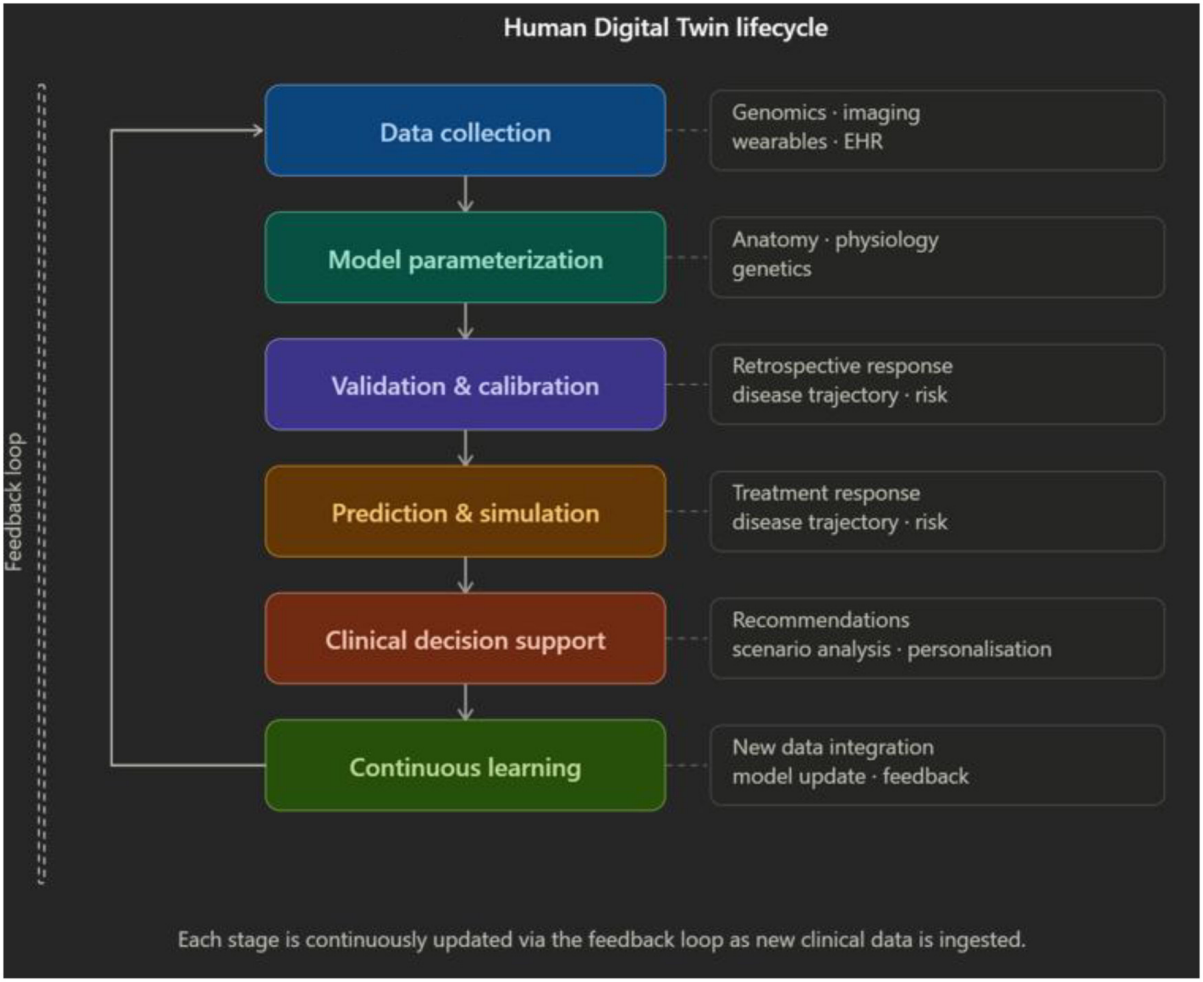
Human digital twin lifecycle. Illustrates the entire operational lifecycle of an HDT, beginning with multi-modal data collection (genomics, imaging, wearables, and EHR) to model parameterization and validation, prediction and simulation, clinical decision support and continuous learning, founded on a feedback loop. The notes on the right define the data inputs and outputs at every stage of the lifecycle, and reveal how HDT operates in an iterative and data-driven manner.

A growing pressure exists to shift away, at the population level, of homogeneous treatments to individualized treatment, taking into consideration the individual biology, genetics, and lifestyle of a patient (1, 2). In fact, advances in genomic sequencing, wearable sensor technologies, electronic health records, and machine learning algorithms have all proceeded pari passu and are aligned to support a paradigm shift toward precision and predictive medicine (3–5). Despite these advances, translating such enabling technologies into demonstrable improvements in patient outcomes remains incomplete, partly because predictive modeling of individual responses to therapeutic interventions prior to clinical deployment is currently insufficient. The concept of a digital twin-virtual representations of physical entities that integrate real-time data with computational models—arose within the fields of manufacturing and aerospace, where the fidelity of simulations directly impacts product quality and safety (6, 7, 35). More recently, this engineering paradigm has attracted growing interest from biomedical researchers and clinicians aiming to develop virtual patient models mimicking individual anatomy, genetic variation, and physiology (8, 9). Unlike classic computational models that are aimed at population-level inference, human patient digital twins operate at the level of the individual; their predictions are in constant flux, updated by novel clinical data in a feedback loop to provide real-time therapy suggestions (10, 11). The complete operational lifecycle of an HDT—from multi-modal data ingestion through model parameterization, simulation, and clinical decision support—is illustrated in Figure 1. The creation of human digital twins represents a paradigm shift from reactive management of manifested disease to proactive prediction and prevention, using computational simulations of individual patient biology (3, 12). In early cardiology, these comprehensive, patient-specific models have shown the ability to predict heart-failure progression, optimize device-implantation strategies, and forecast treatment responses with much greater accuracy than population-level statistical methods achieve (10, 12, 13). Given this trajectory for human digital twins in oncology, immunology, and multi-system disease management, there should be significant opportunities for wide-ranging impacts on precision healthcare delivery (1, 9, 14, 15). This work addresses the challenges of impeding the translation of HDT research into routine clinical practice along technical, regulatory, ethical, and organizational dimensions. The methodological approaches to the validation of predictive and decision-support models are heterogeneous and often insufficient for clinical decision-making settings (11). Interoperability of different data sources, computational platforms, and clinical workflows is only partially achieved (16). Governance frameworks that ensure the ethical deployment of these technologies have not been systematically developed in most healthcare systems to date (17–19).

### 1.1 Novelty and contribution of this review

Several prior reviews have addressed specific aspects of the HDT ecosystem (summarized in Table 1, Section 8). Björnsson et al. (1) focused on genomic integration but did not address regulatory or governance dimensions. Corral-Acero et al. (10) included virtual heart models in their scope, but left out oncology and immunology. Laubenbacher et al. (14) experimented on the digital immune twins, but did not incorporate it into systems. The clinical preparedness and level of evidence were evaluated by Vicesconti et al. (11), but they suggested not many practical solutions. All of the currently published reviews lack a unified approach to include all four domains, i.e., AI methods, multi-domain clinical use (cardiology, oncology, immunology and metabolic medicine), ethical governing models and a roadmap to future research (2025–2035) into a single framework. The existing review addresses this gap by integrating technical, clinical, regulatory and ethical factors in an integrated manner and providing practical suggestions to researchers, clinicians and policymakers (as presented in Table 1).

**Table 1:** Comparative analysis of recent HDT and precision medicine surveys (2019–2025).

Survey	Primary focus	Key contribution	Identified gap
Grieves & Vickers (6)	Foundation DT framework	Systematic DT definition	Limited healthcare specificity
Fuller et al. (20)	DT enabling technologies	IoT, cloud, AI infrastructure	Limited clinical validation
Corral-Acero et al. (10)	Digital twins in cardiology	Virtual heart models	Limited oncology/immunology
Björnsson et al. (1)	HDT personalized medicine	Genomic integration	Regulatory pathway unclear
Liu et al. (8)	Human digital twins	Survey of HDT approaches	Limited critical evaluation
Laubenbacher et al. (14)	Digital immune twins	Immune system modeling	Limited system integration
Viceconti et al. (11)	Clinical readiness	Evidence standards	Limited solutions proposed
Current Survey (2025)	Comprehensive HDT review	Integrated governance frameworks and research roadmap	Future directions outlined

## 2 Review methodology

The four large systematic search strategies were used to conduct a systematic narrative review. Academic databases i.e.,: IEEE Xplore, PubMed, Scopus, and Google Scholar. The search was performed in January 2024 and February 2025 with the focus on the literature that was published since 2016 with the preference to publications dated between 2019 and 2025.

### 2.1 Search keywords

Subsequently, such key phrases were used: human digital twin, digital twin healthcare, virtual patient model, digital twin cardiology, in silico medicine, federated learning healthcare, precision medicine AI, physiological modeling, digital twin oncology and predictive healthcare simulation.

### 2.2 Inclusion criteria

The articles were included because of the following criteria: (i) had to be related to the concept of digital twins in human health or clinical medicine; (ii) had to discuss the enabling technology (machine learning, physiological modeling, or data infrastructure); (iii) had to report clinical use or validation results; or (iv) had to address the ethical, regulatory, or governance implications of the HDT implementation.

### 2.3 Exclusion criteria

The literature was narrowed down to those which: (i) did not involve any healthcare related digital twins or non-biological applications; (ii) were not published as conference abstracts; (iii) were not opinion pieces or editorials that did not contain any empirical or methodological information; or (iv) were not written in English.

After screening of title, abstract and full-text, 35 peer-reviewed articles and technical reports were examined, and tabulated to form the evidencing basis of this survey. This cumulatively is 35 sources is in agreement with the figure mentioned in the abstract. No primary data were collected and the analysis is who completely relies on the published literature found in accordance with the systematic search process outlined above. Keywords used on search, inclusion criteria and selection process is defined in the former subsections.

## 3 Conceptual foundations of human digital twins

### 3.1 Defining digital twins in healthcare context

A digital twin is a high-fidelity, integrated virtual representation of an anatomical, physiological, genetic, and clinical attributes of a specific patient that is continuously updated in near real-time with clinical data, laboratory analysis, imaging and sensor data (6, 8, 20, 33). This definition draws a very clear line between digital twins and other related but different concepts, e.g., electronic health records, which store cumulative clinical data but lack predictive modeling, computational models, which model population averages as opposed to individual patients, and clinical decision support systems, which use population-based algorithms without individualization to patient-specific biometric parameters. The digital twins hierarchy is a hierarchy of biological organization that includes a number of levels of temporal scales (9). On the molecular level, models are constructed based on genomic information, proteomic measurements and metabolomic signatures in order to model drug metabolism, gene expression regulation and molecular pathogenesis of disease. The heterogeneity is provided by the cellular and tissue levels in intra-populations, tissue architecture, and characterization of the microenvironment. Organ-level models are models of integrated physiological processes in terms of coupled differential equations, which describe blood flow, mechanical deformation and electrical conduction, among other processes that are specific to an organ. Models on the system level indicate the relationship of different organs, and the overall processes of physiological regulation (12, 14, 21).

### 3.2 From static models to dynamic virtual patients

**Figure 2:**
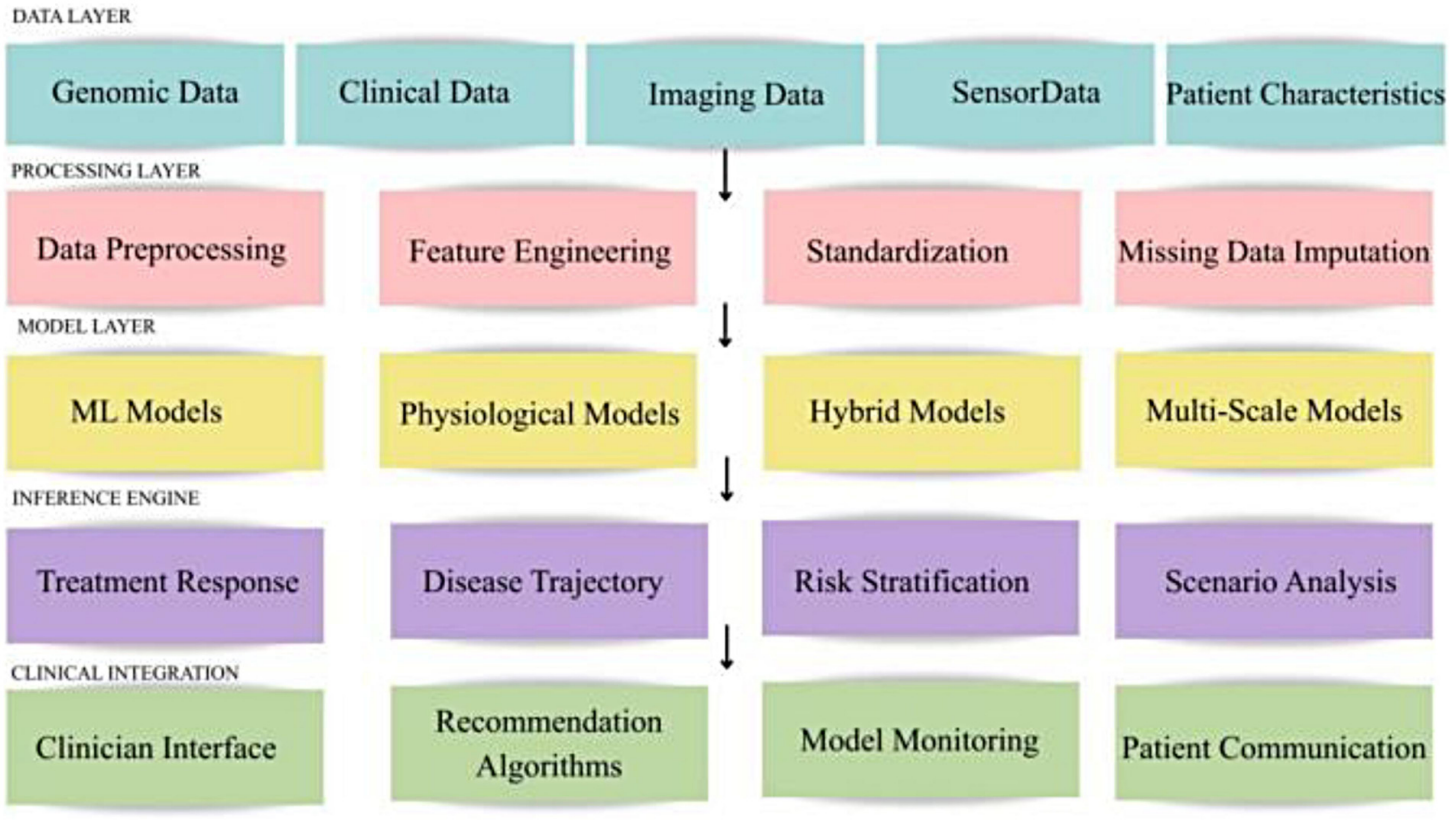
Digital Twin System Architecture. Shows the stacked technical architecture of an HDT system on five levels, namely the Data Layer (genomic, clinical, imaging, sensor, and patient characteristic data inputs), the Processing Layer (data preprocessing, feature engineering, standardization, and missing data imputation), the Model Layer (ML models, physiological models, hybrid models, and multi-scale models), the Inference Engine (treatment response prediction, disease trajectory modeling.

The standard form of presentation of conventional computational models of biomedical research is usually static or quasi-static, capturing disease mechanisms or treatment effects either at discrete timepoints or in time windows (12, 22). On the contrary, the digital twin approach introduces the continuities of processing at a temporal dynamic level, and incorporates feedback loops, nonlinear interactions, and adaptive mechanisms that are typical of a living biological system (8, 9). This time aspect emphasis helps in making predictions with regard to the disease paths. Recognition of key intervention times, and modeling of response dynamics to therapeutic modifications (10, 13). The concept of virtual physiological humans provides historical background to integrated multi-scale modeling approaches that have since been adopted within modern digital twin paradigms (21). Initial virtual human projects focused on fundamental physiological accuracy and model interpretability, establishing proof-of-concept that detailed computational models of human biology were even possible to create (22). Contemporary digital twin methods build on these beginnings to include sophisticated machine learning techniques, real-time data ingestion infrastructures, and direct integration into clinical practice (8, 16). The five-layer technical architecture underpinning this approach is presented in Figure 2.

## 4 Enabling technologies for human digital twins

### 4.1 Machine learning and artificial intelligence

The current digital twin solutions primarily rely on machine learning algorithms that detect and quantify complex, nonlinear relationships among high-dimensional clinical, genomic, and imaging datasets (4, 5, 23). Deep learning architectures have especially great potential for the processing of unstructured data, including, but not limited to, medical images, narratives from electronic health records, and time-series physiological signals (5, 24). Recurrent neural networks and transformer-based architectures effectively model temporal dynamics and sequential dependencies that are inherent in the disease trajectories at the bedside (24). Graph neural networks encode relationships among heterogeneous patient characteristics and disease mechanisms, therefore facilitating integration with multimodal data sources. Machine learning approaches complement mechanistic physiological modeling by learning empirical input–output relationships when first-principles models are insufficiently detailed or computationally intractable (4, 5). Hybrid approaches that merge mechanistic models with machine learning components combine the best from both paradigms: mechanistic models offer interpretability and extrapolation capability, while machine learning components capture complex interactions poorly represented by simplified mechanistic equations (8, 16).

### 4.2 Physiological modeling and computational mechanics

Organ physiology Organ physiology is modeled as patient-specific networks of differential equations of mass transport, energy metabolism, electrical signaling and mechanical deformation (12, 13, 21, 25). The cardiac modeling paradigm is the best paradigm of such a strategy: elaborate virtual heart modeling with consideration of electrophysiology, mechanical contraction, blood flow dynamics, and neurohumoral control can be utilized to forecast susceptibility to arrhythmia, mechanical failure, and device therapy performance (10, 12, 13). The biomechanics modeling is applied to the effect of muscle forces and joint loading to predict the likelihood of musculoskeletal injury and to aid in rehabilitation (25). Pharmacodynamic-pharmacokinetic models are used to describe the pharmacodynamics and pharmacokinetics of some drugs in an isolated patient with respect to the genetic polymorphism of the metabolic enzymes and altered physiology in old age or comorbid disease (4, 22).

### 4.3 Infrastructure for data integration and interoperability

The ability to seamlessly integrate data sources that enable the effective implementation of the digital twin hinges on the integration of the data sources are heterogeneous in nature, such as genomic repositories, imaging archives, wearable sensor laboratory information systems, clinical notes, and networks (16, 26). The use of standardized data formats, ontologies, and application programming interfaces enables automated data extraction and transformation in support of the analytics (16). The scalable computing resources required by detailed physiological simulations and machine learning model training are delivered through cloud computing infrastructure (20, 27). Federated learning architectures can enable collaborative model development across multiple healthcare institutions while guaranteeing data privacy and regulatory compliance by processing sensitive patient data locally and sharing only aggregated model parameters rather than raw data (26, 28).

## 5 Personalized and predictive healthcare applications

### 5.1 Cardiovascular applications

Precision cardiology is the most clinical advanced field of digital twin use, and patient tailored cardiac models have been found to be helpful in a variety of treatment fields (10, 12, 13) (see Table 2 for a summary of clinical applications and implementation status across domains). The virtual cardiac models, which consider patient anatomy of cardiac images, electrical characteristics of electrocardiography and mechanical characteristics of echocardiography, can predict the risk of arrhythmia, optimize implantable devices, and predict patient response to pharmacological and device-based therapies (10, 13).

**Table 2:** Clinical applications of human digital twins.

Application domain	Clinical problem	Digital twin approach	Current evidence	Implementation status
Cardiology	Heart failure progression	Patient-specific cardiac mechanics + ML	Moderate	Research/Pilot
Cardiology	Arrhythmia risk	Electrophysiologic modeling + imaging	Moderate	Research/Pilot
Cardiology	Device optimization	Biomechanical simulation	Moderate	Research/Pilot
Oncology	Treatment response	Genomic + imaging + mechanistic tumor models	Early	Research
Oncology	Clinical trial enrollment	Virtual cohort simulation	Early	Research
Immunology	Vaccine response	Immune system modeling + genetics	Early	Research
Immunology	Infection risk	Multi-pathogen immune models	Early	Research
Metabolic	Diabetes progression	Glucose-insulin dynamics + genetics	Moderate	Research/Pilot
Respiratory	Respiratory disease	Lung mechanics + inflammation models	Early	Research
Neurology	Stroke outcome	Brain physiology + perfusion models	Early	Research

Virtual patient cohorts are developed as aggregates of patient-specific cardiac models that can predict drug efficacy and safety in populations with no need to conduct prospective clinical trials (12). Such a strategy can minimize the development time and cost of new cardiovascular therapies, and also improve trial design by pre-identifying patient subpopulations most likely to benefit or be harmed (12, 22). Nevertheless, clinical validity of these virtual cohorts will depend on how representative and good the training data are to parameterize individual models.

### 5.2 Oncology and genomic medicine

**Figure 3:**
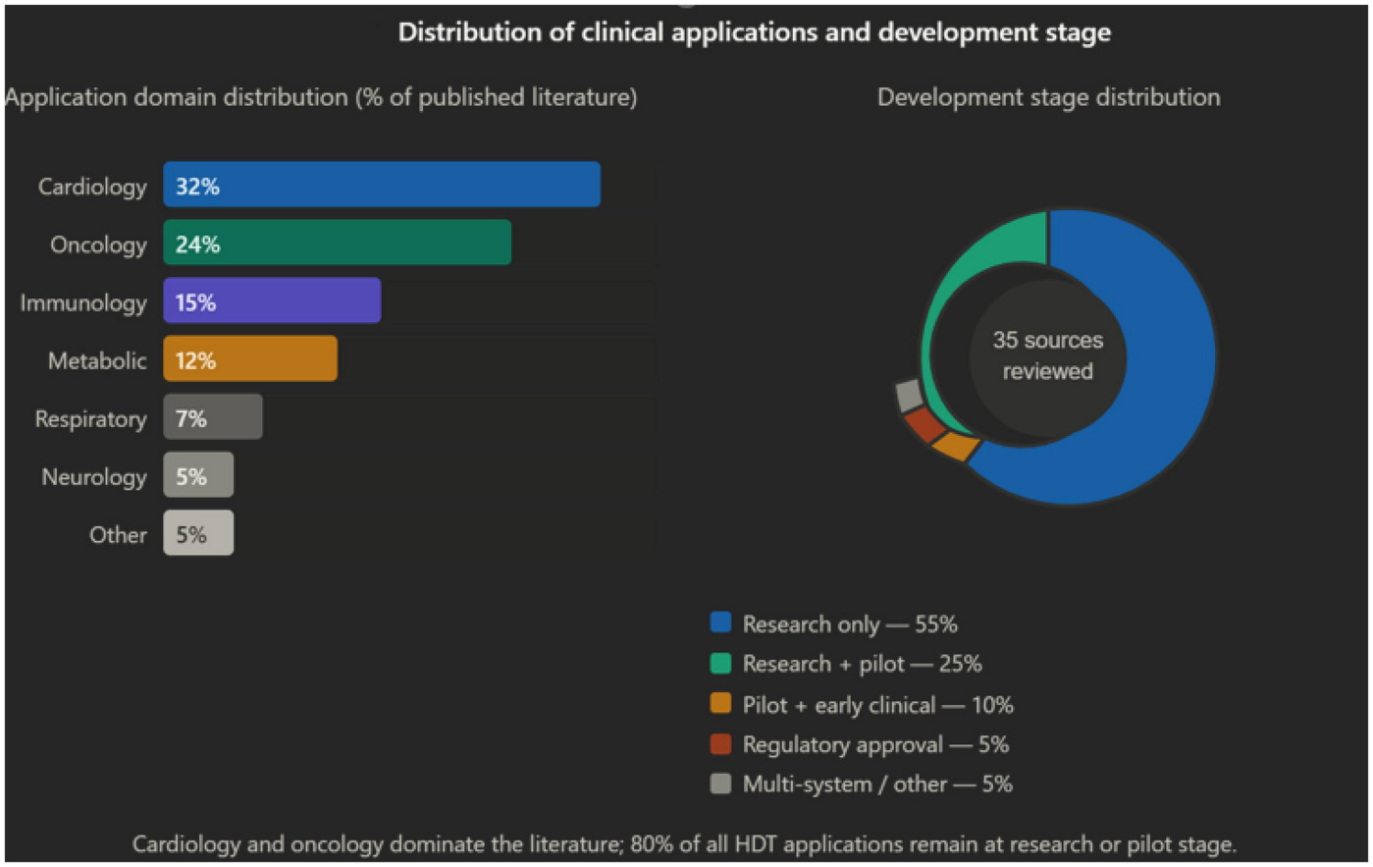
Distribution of Clinical Applications and Development Stage. Provides two charts that describe the landscape of HDT clinical applications. The left bar chart (Application Domain Distribution) illustrates the relative size of the published literature in terms of clinical domains with cardiology and oncology taking the largest part of contributions. The right chart (Development Stage Distribution) shows the percentage distribution of HDT applications by stage of maturity: 55% are still in the research-only stage, 25% are in the research-plus-pilot stage, 10% are in the pilot/early clinical stage and 5% are in the regulatory approval stage, indicating the overall early-stage nature of HDT clinical translation.

Cancer is fundamentally a heterogeneous disease, the reaction to treatment and prognosis of a specified tumor is predetermined by the biology of a specific tumor (1, 15, 29). The distribution of HDT applications across clinical domains, including oncology, is presented in Figure 3. Digital twin algorithms combine tumor genomic sequencing, tumor functional imaging properties, patient germline genetics, and immune profiling to predict response to targeted therapy, immunotherapy, and combination therapy (1, 15, 16). Tumor-immune interactions are analyzed analytically by means of a mechanistic approach in order to assist in the determination of the best dose and sequencing of immunotherapy (14, 29).

Model groups of patients may be designed specifically with respect to the development of tumors in conjunction with other treatment modalities. There are early signals that algorithms that are based on population averages may be defeated by simulation-informed therapy choice (1, 15), but no prospective trial has succeeded to establish the efficacy of simulation-based therapy choice in a general oncological setting.

### 5.3 Immunology and infectious disease

Digital twins incorporating immune system modeling enable prediction of vaccine response, risk of severe infections, and effectiveness of immunomodulatory therapies (9, 14). Models capturing patient-specific immune repertoire, prior pathogen exposures, and genetic determinants of immune function inform personalized immunization strategies and predict risk of vaccine non-responders who require alternative approaches (14, 29).

Terminology Early Early in pre-clinical or even earlier phases of research in computational research, or with little prospective clinical validation. Moderate The applications whose pilot or feasibility clinical data have already been reported but not proven by a prospective trial in large scale. Research/Pilot: shows that evidence-of-concept studies or mini-clinical pilot studies are already done, but the regulatory approval or regular clinical utilization has not been reached. According to studies, work is limited to the computational or lab settings.

## 6 Clinical use cases and virtual cohorts

### 6.1 Virtual patient cohorts in drug development

Conventional drug development has strongly depended on clinical trials that involve thousands of patients and in long periods of time at high costs (12, 16, 22). Virtual patient cohorts are groups of heterogeneous digital twins, reflecting the heterogeneity of human populations, that can be used to predict drug efficacy and safety in patient subgroups in silico (12, 22), increasing the drug development rate, eliminating the necessity to recruit real patient groups, and predicting patient groups that are likely to respond or be toxic (6).

Nevertheless, even with these strengths, the application of virtual cohorts in regulatory filings is now constrained by a lack of validation data and ambiguity about evidentiary standards (11, 16). Regulatory mechanisms that can be used to help bridge the gap between in silico evidence and traditional trials are underway, but there is yet no consensus on reasonable validation criteria (16, 22, 30).

### 6.2 Precise treatment planning in heart failure

Patients with advanced heart failure have a poor quality of life and limited survival despite optimal medical therapy, thus providing a very strong rationale for a personalized approach to advanced therapies, such as ventricular assist devices and transplantation (10, 12, 13). A patient-specific cardiac digital twin, including detailed anatomy from cardiac imaging, incorporating electrical properties from electrocardiography, and hemodynamic characterization, allows virtual implantation of candidate devices and the simulation of mechanical and electrical consequences (10, 12, 13). Preliminary studies have given indication that simulation-guided optimization of device parameters improves outcomes compared to conventional approaches, offering better preservation of cardiac function and a reduced arrhythmia burden (10, 13).

## 7 Ethical, privacy, and trust considerations

### 7.1 Data privacy and regulatory compliance

Since digital twin applications intrinsically involve the processing of enormous amounts of personal health data, this also results in significant privacy risks and regulatory burdens pertaining to healthcare privacy regimes such as HIPAA, GDPR, and emerging AI-specific laws and regulations (17, 18, 26, 28). The emerging approach for circumventing these privacy constraints involves federated learning architectures that train models from decentralized data by preserving privacy in the originating healthcare systems (26, 28). However, their implementation requires significant levels of technical infrastructure and governance mechanisms necessary for ensuring appropriate oversight (26, 28). Complementary approaches for privacy protection include synthetic data generation and differential privacy techniques, but this generally involves a trade-off between strength of privacy guarantees and model performance (16, 26, 28).

### 7.2 Algorithmic bias and health equity

Machine learning algorithms trained on non-representative populations exacerbate existing healthcare disparities through biased predictions that affect members of under-represented groups (5, 23, 26). Digital twins incorporating patient-specific genetics and physiology rather than using race or ethnicity as proxies for biological variation are an improvement over the conventional approach of population stratification, but nonetheless require active strategies for the collection of diverse training data and evaluation for bias (5, 16, 26). Transparency, Explainability, and Trust Clinicians and patients need to understand digital twin recommendations, so that they can accept or be skeptical of the predictions (16, 17, 19). Black-box machine learning models have limited interpretability, which creates a significant barrier to clinical acceptance and regulatory approval (5, 16, 19). Explainable AI techniques, such as attention mechanisms and saliency mapping, and the incorporation of mechanistic models, provide increased transparency at the possible cost of reduced predictive performance (5, 16, 19).

### 7.3 Transparency, explainability, and trust

**Figure 4:**
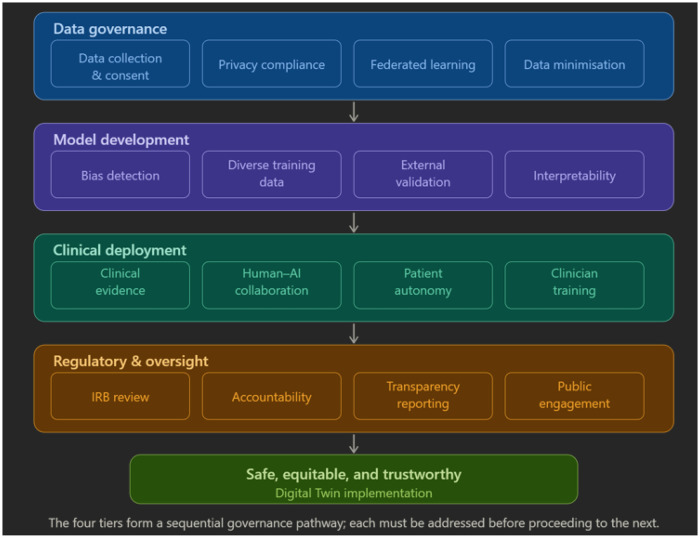
Ethical governance framework for digital twin deployment. Presents a structured four-tier ethical governance framework for HDT deployment The layers target: (1) Data Governance, such as the data collection and consent, adherence to privacy, federated learning, and data minimization, (2) Model Development, such as the need to identify bias, training data diversity, external validation, and interpretability, (3) Clinical Deployment, such as the need to have clinical evidence, human-AI collaboration, patient autonomy, and train-the-clinician, (4) Regulatory and Oversight, specifying IRB review, accountability, transparency reporting, and public engagement. The framework converges on the goal of safe, equitable, and trustworthy digital twin implementation.

Clinicians and patients need to understand digital twin recommendations in order to appropriately accept or critically evaluate model predictions (16, 17, 19). A structured ethical governance framework addressing these considerations across four tiers is presented in Figure 4. Black-box machine learning models have limited interpretability, which creates a significant barrier to clinical acceptance and regulatory approval (5, 16, 19). Explainable AI techniques—such as attention mechanisms and saliency mapping—and the incorporation of mechanistic models provide increased transparency, though potentially at the cost of reduced predictive performance (5, 16, 19).

## 8. Comparative analysis with existing surveys

Table 1 gives a systematic comparison of seven major previous surveys to the current review. This comparative discussion describes the evolving process of literature on HDT where a theoretical ground has been substituted with an emphasis on clinical translation. The previous surveys focused more on technology facilitators and the more recent surveys emphasize more on the rise of attention to rigor of validation, ethical governance and regulatory pathways. There is no complete standardization of validation methodology, interoperability standards and integrating evidence frameworks.

## 9 Open challenges and research gaps

### 9.1 Standards for model validation and clinical evidence

The literature on digital twins is characterized by various validation approaches that ascertain stringent evidence synthesis with regards to its predictive performance and clinical usefulness (11). There are limited future clinical trials of digital twins-guided interventions, and the majority of the evidence is founded on retrospective analysis or even computer simulations (11, 16, 30). Standardization is the key priority, which has to be in the form of multilevel validation frames comprising of computational accuracy, clinical predictive validity and outcome effectiveness (11, 16, 30). Research-grade models, which are developed to produce a hypothesis and clinical-grade models, which need to be employed to produce informed patient-care, are distinctly different decisions (11, 16). Majority of the regulatory systems in various jurisdictions are not well established but even to state the evidentiary standards and routes to the approval of Use of digital twins in a clinical environment (11, 26, 30).

### 9.2 Data integration and interoperability

Health systems have heterogeneous information infrastructures with incompatible information data formats and little integration of clinical and research data silos, and patient data there is a divided ownership among institutions (16, 26). Agreement on data standards, adopting safe measures of interoperability of data, and creating regulation technological and organizational demands of mechanisms that permit the sharing of data in a controlled way are daunting challenges (16, 26).

### 9.3 Multiscale integration and model complexity

Digital twins of molecular, cellular, tissue, organ and system-scale processes whole-system have large computational cost, which requires new algorithms to shrink model, quantifying uncertainty and good computing (16, 31). In the practice to clinical circumstance, the trade-off between comprehensiveness of the model and computational tractability needs to be explicitly considered (16, 31).

### 9.4 Ethical governance and equity

Ethical scrutiny procedures should be offered in the future governance structures in a meaningful way intervention of the risk communities, and preventive health measures inequities (17–19, 26, 30). The existing bioethics and research governance infrastructure offers little explanation of the problems that are particular to the implementation of digital twins in practice (17, 26, 30).

## 10 Future directions

### 10.1 Foundational research priorities

**Figure 5:**
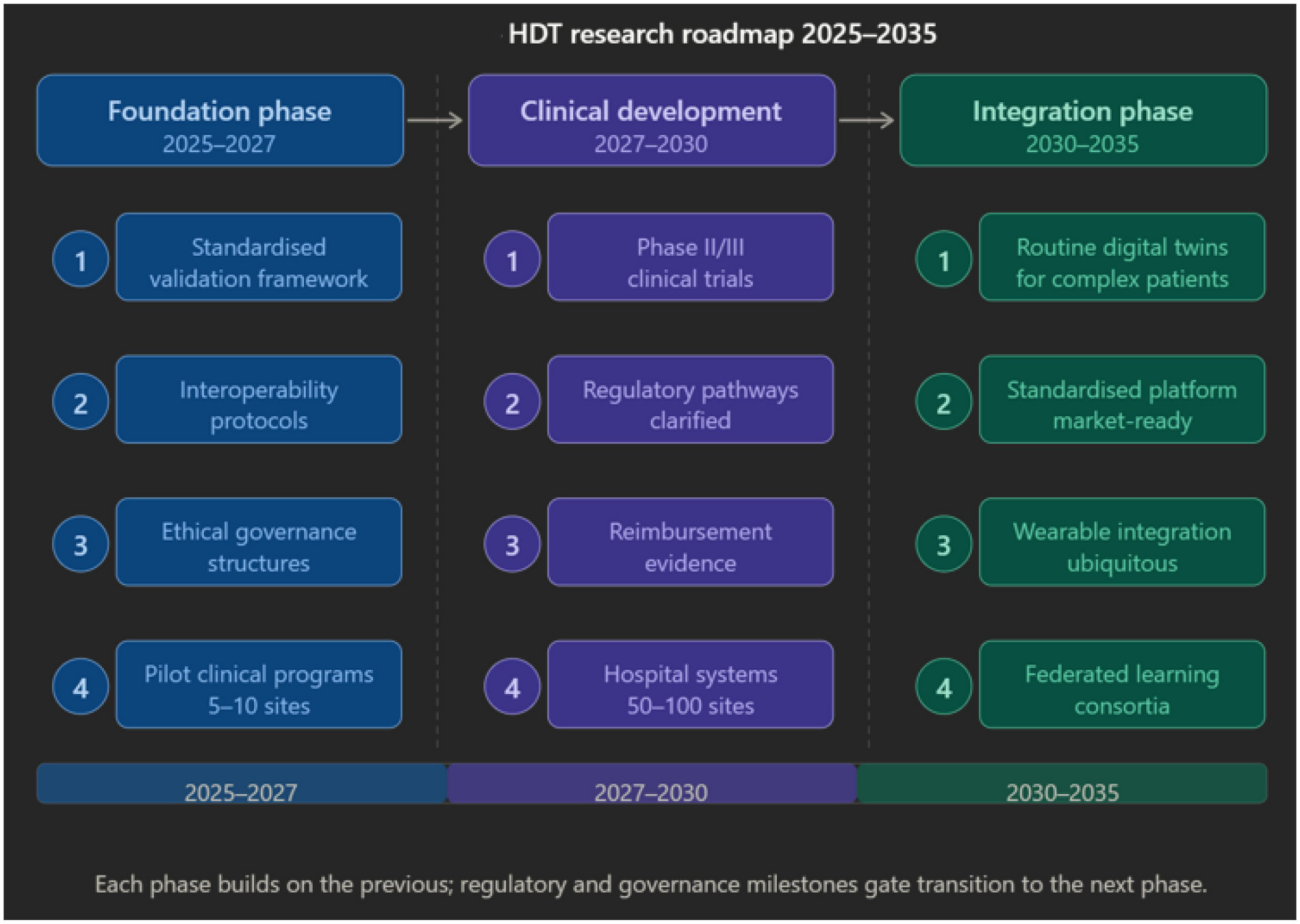
HDT research roadmap 2025–2035. Gives a three stage HDT research and implementation roadmap. Phase 1 The focus is put on standardized validation frameworks, interoperability (Foundation Phase, 20252027). protocols, ethical governance structures and pilot clinical programs with 5–10 sites. Phase 2 (Clinical Development Phase, 20272030) is targeted at Phase II/III clinical tests, regulatory clarification, trails, building of reimbursement evidence, and growth to 50–100 hospital systems. Phase 3 The imaginative (Integration Phase, 20302035) envisions ordinary digital twins of complicated patients, standardized market ready systems and ubiquitous wearable integration and federated learning consortia at scale.

The development of digital twins science needs long-term investment in model validation procedures, and uncertainty quantification models (16, 26, 30, 31). A three-phase research roadmap that summarizes these priorities is given in Figure 5. Adaptation of new data formats—such as spatial transcriptomics, new functional imaging, and wearable sensor longitudinal data—offers new possibilities of model improvement and predictive capability (9, 16, 29, 31, 32). One area of research is especially significant: The creation of explainable artificial intelligence approaches that are well blended with mechanistic physiological models, enabling clinical translation (5, 16, 19, 26). In digital twins, demonstration projects have been carried out. Implemented into the daily practice of clinical work, carried out in cooperation with the work of Academic Medical Centers, Regulatory agencies, and the industry, can possibly develop evidence on clinical utility, cost effectiveness, and implementation feasibility (16, 26, 30, 31). High-need clinical area pilot projects advanced heart failure, precision oncology, and immune compromised. Patient care-represent especially opportune targets (16, 26, 30). Future healthcare ecosystems are expected to evolve toward human-centric intelligent systems aligned with the principles of Industry 5.0 (34).

## 11 Conclusion

Human digital twins are the best site of convergence of the goals of precision medicine and speed progress in computational and data-analytic skills, which have giant potential in the field of individualizing health care, foreseeing illness, and expedited treatment development. First cardiology exhibits and new applications in. oncology immunology show clinical feasibility and improvements in treatment individualizing as compared to population-average strategies. However, research translation to everyday clinical practice does homage to gigantic unresolved problems in the technical dimensions, including model validation, interoperability, and create clinical evidence, regulatory approval procedures, and social aspects like ethical governance, health equality, and public engagement. A single solution cannot be found to these multidimensional issues areas of work or within one organization in isolation; rather, they require a concerted effort collaboration between academia, industry, healthcare systems, regulatory agencies, and patient communities to develop digital-twin science to clinical integration. The priority research investments have to be made in coming up with standard validation structures which will be in a position to allow high-quality assessment of digital twins quality and clinical use, interoperability procedures that enable safe integration of information among health systems, and moral administration designs that ensure proper supervision and just enforcement. Simultaneously, the development of regulative routes and payment proof will be exemplary of financial feasibility and feasibility in terms of implementation. With continued commitment to the challenges and policy emphasis on equity, transparency, and public engagement, digital twins have the potential to reshape health care over the next decade from reactive treatment of disease once it is manifest to proactive personalized predictive medicine that optimizes health outcomes with respect for individual autonomy and diverse values. A historic opportunity for transforming healthcare due to convergence of technological capability with clinical need now exists; realization of that potential necessitates coordination of action beginning without delay.
